# A bioclimatic characterization of high elevation habitats in the Alborz mountains of Iran

**DOI:** 10.1007/s00035-018-0202-9

**Published:** 2018-02-06

**Authors:** Jalil Noroozi, Christian Körner

**Affiliations:** 1 Department of Botany and Biodiversity Research, University of Vienna, Rennweg 14, 1030 Vienna, Austria; 2 Institute of Botany, University of Basel, Schönbeinstrasse 6, 4056 Basel, Switzerland

**Keywords:** Alpine ecology, Biogeography, Microclimate, Treeline, Soil temperature, Vegetation type

## Abstract

The Alborz mountains in N-Iran at 36° N rise from the Caspian Sea to 5671 m a.s.l., with warm-temperate, winter-deciduous forests in the lower montane belt in northern slopes, and vast treeless terrain at higher elevation. A lack of rainfall (ca. 550 mm at high elevations) cannot explain the absence of trees. Hence, it is an open question, which parts of these mountains belong to the alpine belt. Here we use bioclimatic data to estimate the position of the potential climatic treeline, and thus, define bioclimatologically, what is alpine and what is not. We employed the same miniature data loggers and protocol that had been applied in a Europe-wide assessment of alpine climates and a global survey of treeline temperatures. The data suggest a potential treeline position at ca. 3300 m a.s.l., that is ca. 900 m above the upper edge of the current oak forest, or 450 m above its highest outposts. The alpine terrain above the climatic treeline position shows a temperature regime comparable to sites in the European Alps. At the upper limit of angiosperm life, at 4850 m a.s.l., the growing season lasted 63 days with a seasonal mean root zone temperature of 4.5 °C. We conclude that (1) the absence of trees below 2850 m a.s.l. is clearly due to millennia of land use. The absence of trees between 2850 and 3300 m a.s.l. is either due to the absence of suitable tree taxa, or the only potential regional taxon for those elevations, *Juniperus excelsa*, had been eradicated by land use as well. (2) These continental mountains provide thermal life conditions in the alpine belt similar to other temperate mountains. (3) Topography and snow melt regimes play a significant role for the structure of the alpine vegetation mosaics.

**Electronic supplementary material:**

The online version of this article (10.1007/s00035-018-0202-9) contains supplementary material, which is available to authorized users.

## Introduction

High elevation ecosystems have to be viewed from a climatological, rather than an elevation perspective, because the driver of life is the microhabitat’s climate rather than meters above sea level (Körner 2011). Temperature is a key climatic factor which sets rather narrowly defined growth-physiological limits to plant life at high elevation such as that for alpine treelines (Körner and Paulsen 2004). However, for small stature plants, climatological data from weather stations are not very useful because these plants decouple themselves from free atmospheric conditions and produce their own microclimate (Scherrer and Körner 2010, 2011). Top soil temperatures, that is where most alpine plants have their apical meristems, come very close to the effective alpine bioclimate and are most useful for standardized comparisons between different alpine habitats (Körner et al. 2003). In fact, such soil temperatures could be biologically more relevant for plants than air temperature (Pregitzer et al. 2000). In addition to buried shoot meristems, roots are very sensitive to soil temperature in cold environments (Alvarez-Uria and Körner 2007). It had been shown that the microscale distribution of alpine taxa is related to soil temperature (Scherrer and Körner 2011).

In open terrain beyond the regional tree limit, the micro-habitat variation of surface and soil temperature is mostly driven by micro-topography and slope orientation (Scherrer and Körner 2010). Local topography in particular, has been shown to have a strong influence on the extremes and dynamics of alpine soil temperatures (Wundram et al. 2010; Graham et al. 2012). In alpine terrain, topography creates a mosaic of thermal niches that allow ‘subalpine’, ‘alpine’ and ‘nival’ species to co-occur at short distance (Scherrer and Körner 2011; Graham et al. 2012). To capture the life conditions in such thermal niches, miniature data loggers have been successfully employed (Körner et al. 2003). When such devices are buried in deep shade under high elevation trees or shrubs at 10 cm depth (no radiation driven soil heat flux), convective exchange between air and soil cause such data to mirror weekly means of air temperature as they act on tree crowns (Körner and Paulsen 2004). Hence with such sensor placement, one can also capture atmospheric conditions without installing a weather station.

**Fig. 1:**
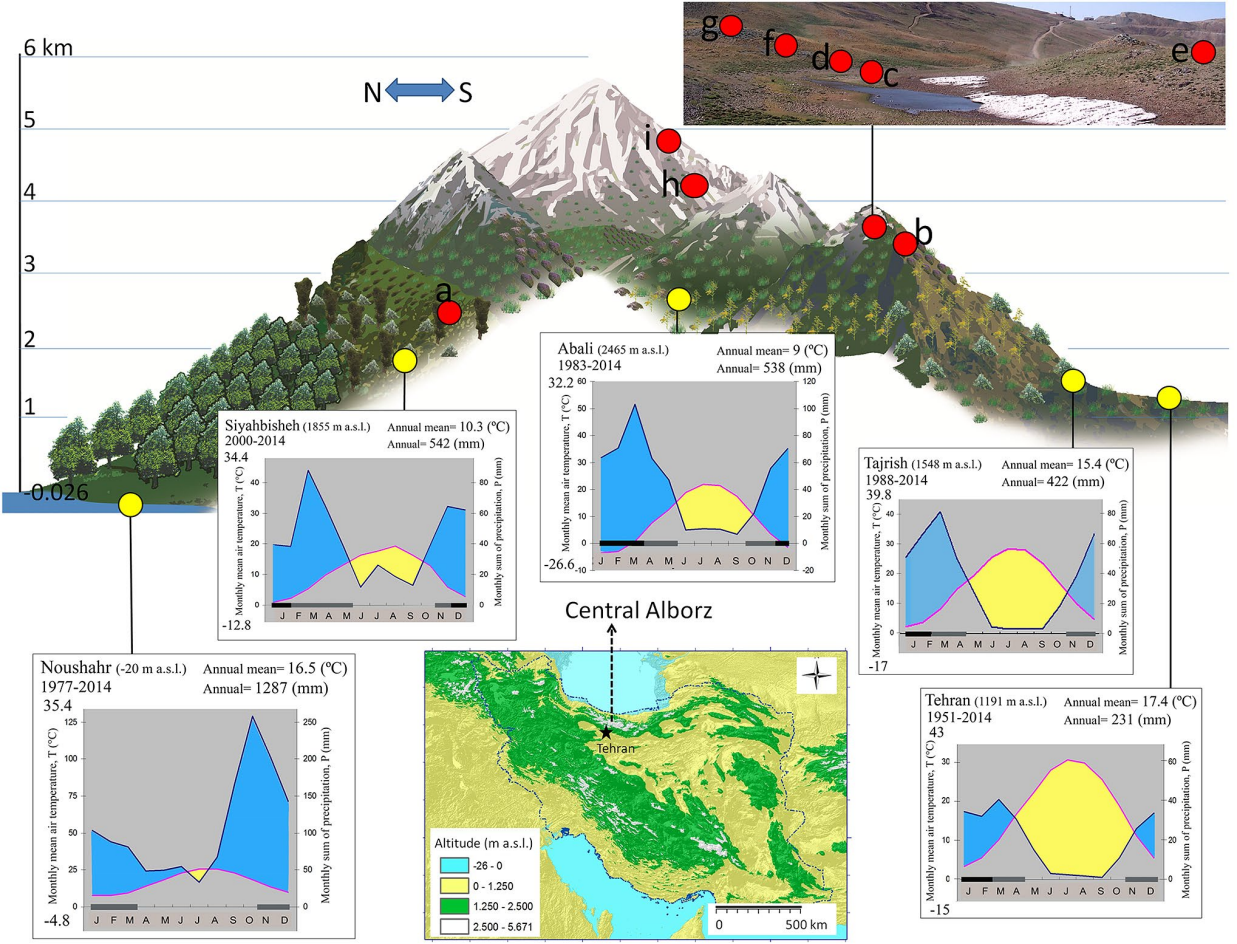
The climate of the Central Alborz based on regional weather station data. Note the aridity gradient from north to south (see also Noroozi 2014). The letters refer to the positions of our data logging campaign (see Table 1)

Here, we employed this technique in different elevations and vegetation types in the Alborz to explore the actual life conditions plants experience, and to compare these to other mountain systems. A specific challenge was to explain the upper limits of trees in these mountains. In such continental mountain systems, one would expect evergreen conifers, the genus *Juniperus* in particular, to form a climatic treeline (as is the case for instance in the Hindukush or in the mountains of Tibet, Miehe et al. 2006). In contrast, the highest elevation forests of the Alborz, which occur on northern slopes only (the Caspian Sea side), are dominated by the deciduous *Quercus macranthera*, commonly reaching up to 2400 m a.s.l., exceptionally, and only in small sheltered pockets, up to 2850 m a.s.l. (Klein and Lacoste 1989; Naqinezhad and Esmailpoor 2017), with a local presence by *Acer hyrcanum, A. platanoides, Fraxinus excelsior, Viburnum lantana*, and *Sorbus aucuparia* (see Table S1). Except for *Sorbus*, these taxa are not known to reach the climatic treeline (a life form limit). Phytogeographically, these forests belong to the Euro-Siberian region (Zohary 1973), and based on a bioclimatic classification, the region belongs to the Mediterranean pluviseasonal-oceanic bioclimate, using the approach by Rivas-Martinez (Djamali et al. 2011). The southern slopes are mostly treeless, as result of the more continental climate and strong insolation during a long dry summer, although weather station data suggest that winter-precipitation still reaches ca. 550 mm > 2500 m a.s.l. (Fig. 1) allowing the scattered existence of woodlands of *Juniperus excelsa*.

**Table 1:** A comparison of canopy-level air temperature (a1) and concurrent soil temperature (a2) in *Quercus macranthera* at the upper limit of oak woodlands, as well as soil temperatures under treeless vegetation at higher elevations (b–i) in the Central Alborz. Temperatures as means or extremes in °C (temperature differences in K)

Site	a1	a2	b	c	d	e	f	g	h	i
Elevation (m a.s.l.)	2406	2406	3475	3690	3693	3695	3662	3710	4212	4850
Slope direction	SW	SW	S	–	SW	–	SW	SW	S	S
Slope (°)	30	30	25	0	10	0	25	10	15	30
Start (DD.MM.YY)	03.06.14	03.06.14	03.09.08	13.08.15	13.08.15	13.08.15	13.08.08	13.08.08	13.08.15	13.08.15
End (DD.MM.YY)	02.06.15	02.06.15	02.09.11	12.08.16	12.08.16	12.08.16	12.08.11	12.08.11	12.08.16	12.08.16
Abs. minimum	− 13.9	− 0.9	− 4.8	− 0.1	− 0.7	− 0.1	− 7.6	− 13.9	− 12.1	− 18.3
Abs. maximum	32.4	17.7	30.7	22.8	16.8	24.3	27.7	27.8	17.1	13.6
Amplitude (K)	46.3	18.6	35.5	22.9	17.5	24.4	35.3	41.7	29.2	31.9
Seasonal mean	13.3	11.3	14.5	10.0	8.1	11.2	11.3	10.9	6.5	4.5
Seasonal median	13.7	12.2	15.2	10.1	8.7	11.6	11.8	11.1	6.7	4.7
Warmest month minimum	14.3^a^	14.7^a^	15.2^c^	9.4^a^	8.8^c^	11.7^c^	12.8^c^	14.7^c^	5.3^c^	1.1^c^
Warmest month mean	18.1^a^	15.4^a^	20.7^c^	14.4^a^	11.3^c^	15.5^c^	17.4^c^	18.2^c^	9.2^c^	5.2^c^
Warmest month maximum	23.4^a^	16.4^a^	27.1c	19.9^a^	14.3^c^	20.1^c^	23.7^c^	22.2^c^	13.3^c^	9.2^c^
Coldest month mean	− 0.2^b^	− 0.1^b^	− 0.4^d^	0.0^b^	0.0^b^	0.0^b^	− 4.5^b^	− 8.3^b^	− 7.9^b^	− 12.4^b^

**Fig. 2:**
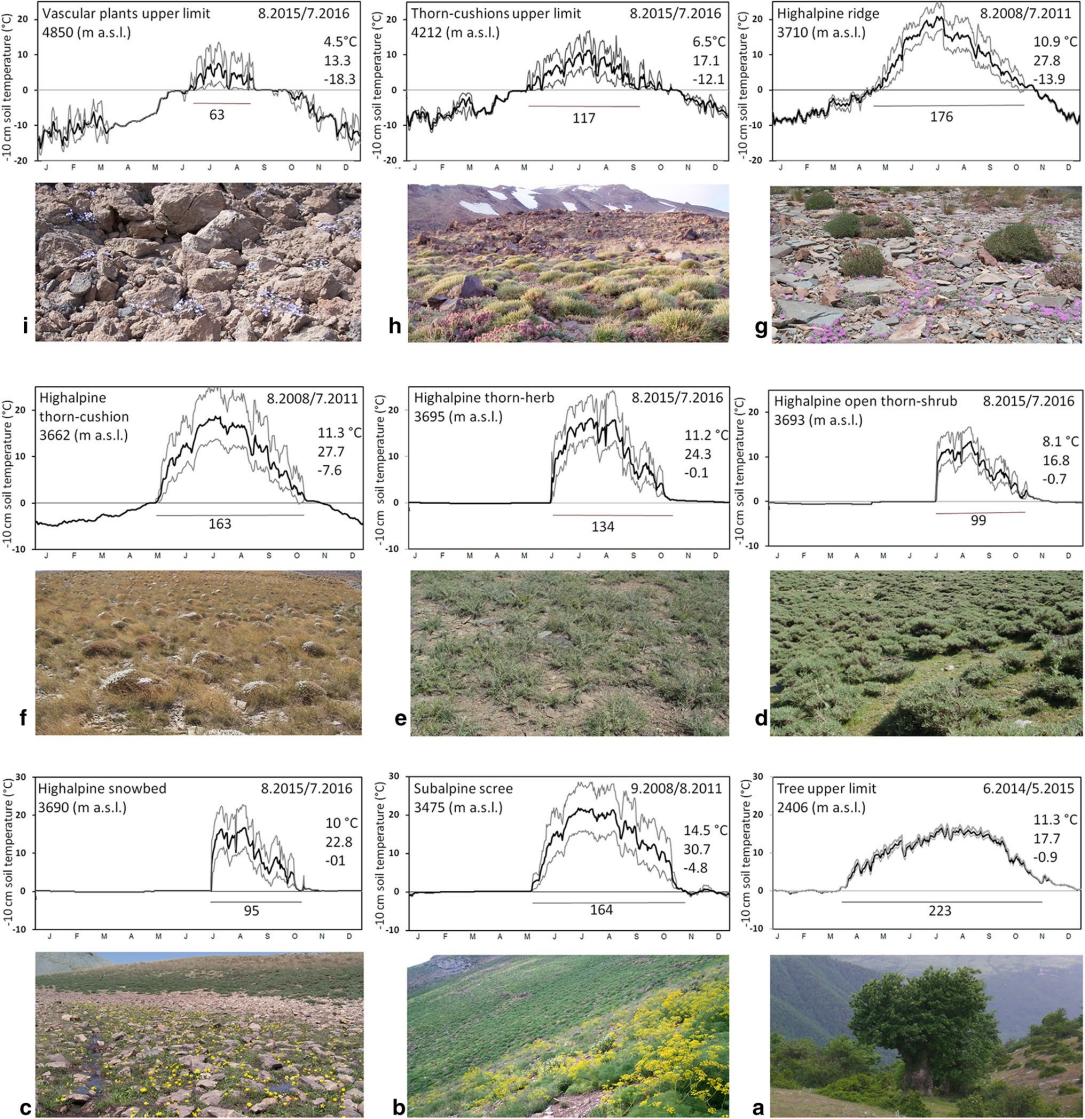
The seasonal course of daily mean − 10 cm soil temperature under short stature vegetation at each of nine sites from close to the potential treeline to the upper limit of vascular plant life. Diagram b, f and g show means for 3 years, all other diagrams are for 1 year. Numbers in the top right corner refer the seasonal mean temperature, and the absolute minimum and maximum. The season length in days, defined by temperature thresholds, is indicated below each diagram. For detail information and the name of each vegetation type see Table 1, and for the locations see Fig. 1. Note, the sites are arranged from highest (top) to lowest (bottom)

The data of sites a1 and a2 are means for two measuring locations at similar elevations each. For locations b, f and g, the data represent means for three years. For the individual years see Fig. S3. For other sites, the data are for one year. For locations see Fig. 1, and for soil temperature diagrams and photographs of vegetations types see Fig 2. The vegetation types and the dominant species of the communities: (a) upper limit of oak woodlands (*Quercus macranthera*); (b) subalpine scree (*Prangos uloptera*); (c) highalpine snowbed (*Ranunculus crymophilus*); (d) highalpine thorn-shrub (*Astragalus jodotropis*); (e) highalpine thorn-herb (*Cousinia multiloba*); (f) highalpine thorn-cushion (*Acantholimon demawendicum*); (g) highalpine ridge (*Jurinella frigida*); (h) thorn-cushion upper limit (*Astragalus macrosemius*); (i) vascular plants upper limit (*Didymophysa aucheri*)

^a^August; ^b^January; ^c^July; ^d^December

Since the upper limit of the life form tree (the treeline) is associated with a globally common isotherm of the seasonal mean temperature (Körner and Paulsen 2004; Körner 2012; Paulsen and Körner 2014), on-site temperatures can help understanding how far the current species-specific tree limits are away from the potential treeline, from where trees might have become lost or never established for unknown reasons. The region is relatively dry (around 550 mm precipitation in the surrounding high elevation weather stations; Fig. 1) but receives far more precipitation than had been found to be prohibitive for high elevation trees (< 250 mm, Paulsen and Körner 2014). In fact, treelines were found to reach particularly high elevations in continental regions with precipitation as low as ca. 300 mm such as in Bolivia or Tibet (Körner 2012). Hence this micrometeorological survey should contribute to the explanation of the absence of trees at the expected high elevation treeline in these mountains. Since the climatic treeline also defines the lower limit of the alpine belt (Körner 2012), these data will also contribute to distinguish the alpine zone of Alborz.

We selected locations at the upper edge of forests, in what might be addressed as the subalpine belt, in alpine snowbeds, meadows, cushion and thorn-cushion vegetation (Noroozi et al. 2010), including their upper limit (Noroozi et al. 2014), and at the upper limit of vascular plants (Noroozi et al. 2011), and we measured the soil temperature under vegetation. We expected that similar soil temperatures will result in similar vegetation types and that the temperature at the upper limit of vascular plants in the European mountains and the Central Alborz are similar. The tree taxa currently reaching highest elevations in the northern Alborz do not belong to species known to be able to grow at the climatic treeline. Therefore, we expect that these species grow at temperatures well above those known for the potential treeline, thus pointing at the absence of treeline taxa in that region, as had been shown for other regions (Körner 2012).

## Materials and methods

### Study area

The mountains of the Central Alborz are in the north of Iran (35° 50′ to 36° 40′ N, 50°50′ to 52°50′ E), representing the highest mountain system of the region, rising sharply from the Caspian Sea level at 26 m below sea level up to the highest summit of 5671 m a.s.l. at Damavand peak. The northern slopes of the Central Alborz receive high amounts of precipitation (between 500 and 1500 mm per year) and would naturally be covered by closed Hyrcanian forests from the Caspian coast up to ca. 2400 m a.s.l., with some outposts recorded up to 2850 m a.s.l. These forests are warm-temperate, winter-deciduous, and contrast European warm-temperate forests by a number of thermophilous Arcto-Tertiary relict species (Akhani 1998). The upper limit of these forests is dominated by *Quercus macranthera* (Klein and Lacoste 1989). The upper limit of vascular plants is reached at ca. 4850 m a.s.l. in Damavand Mt. (Noroozi et al. 2011).

From north to south, across the Alborz, the climate changes from humid warm temperate near the Caspian Sea to continental semiarid, with summer drought increasing dramatically south of the divide (Fig. 1). At higher elevation, the climate and precipitation regime are still of ‘Mediterranean-type’ but with substantial amounts of snow in winter. So, precipitation is depending on both the latitudinal position and the elevation of a site (Khalili 1973; Noroozi et al. 2008). At elevations > 1500 m, the majority of the annual precipitation falls as snow in late autumn, winter and early spring (Fig. 1). Hence, deep soil moisture reservoirs and topography play a critical role for water supply in late spring and summer. The largest glacier of Iran is located in the Central Alborz, on Alamkuh Mountain (4850 m a.s.l) which is very close to our study area. The low edge of this glacier is at ca. 3800 m a.s.l. (Ferrigno 1991).

### Placement of temperature loggers

A standardized and well defined placement of such devices is critical to obtain comparable data. We buried single channel temperature loggers (Tidbit; Onset Computer Corporation, Cape Cod, MA, USA; − 30 to + 70 °C, 0.2 K resolution, 3 cm diameter, 1.5 cm thickness) that have a good record of robustness accuracy at − 10 cm soil depth, following the protocols of (Körner et al. 2003; Körner and Paulsen 2004; Körner 2011). The loggers were programmed to record temperatures at hourly intervals and they were calibrated in ice water for zero degree before and after in situ exposure. In open terrain, loggers were preferentially placed under closed vegetation and on level ground, unscreened by rocks. To assess life conditions of trees, we buried loggers in the deepest possible shade under trees, and placed another set of loggers in the deepest possible shade inside the canopy (under major branches to obtain absolute minima). A 10 cm soil depth provides enough micro-scale spatial integration and buffer against short term (e.g. in the case of small sun-flecks in open terrain a shallower position could bias the readings). The depth is also biologically relevant because this is where most roots occur (Körner and Paulsen 2004). The data for the upper limit of tree species is for a full year from summer 2014 to summer 2015, and for alpine habitats from summer 2015 to summer 2016. Since three loggers in important locations did not work, we adopted data collected with similar devices and protocol from the long-term series of GLORIA sites (GeoPrecision MLog5W loggers, see http://www.gloria.ac.at) from the same area. Hourly data for these loggers were from summer 2008 to summer 2011, exactly three years data.

The lowest loggers were exposed at the upper limit of the Hyrcanian forests at 2400 m a.s.l., the next highest at the upper limit of the subalpine zone at 3475 m a.s.l. in tall herb-fields dominated by umbelliferous plants, and several loggers were buried across the high alpine zone at ca. 3700 m a.s.l. at locations differing in snow-melt regimes. The loggers were placed midway the elevational range of each vegetation type. These latter loggers should reveal topography effects on soil temperature and snow-free season. Finally, we collected data at 4212 m a.s.l., where thorn-cushions reach highest elevations, and at the upper limit of vascular plant life at 4850 m a.s.l. As plants become rare towards their high elevation limits, the exact range limit is often difficult to estimate but the highest vascular plant individuals we could find grew at Damavand Mt. (4850 m a.s.l.), and the logger was placed exactly at this elevation near *Veronica aucheri*.

### Data analysis

Absolute minimum and maximum temperatures, and seasonal mean temperature were used to characterize the thermal regime at each site. The beginning of the growing season was defined by the usually obvious and sharp warming of the ground after snowmelt. In all loggers from open terrain, the first and last incidence of a temperature above + 2 °C, matching the protocol of Körner et al. (2003) was applied. For loggers in deep shade under trees we used the + 3.2 °C threshold that coincides with a weekly mean air temperature of 0 °C (Körner and Paulsen 2004). The choice of these thresholds has very little influence on the resulting season length, because the transitions are rather rapid. The mean and median for the growing season defined by these thresholds, minimum, mean and maximum values of warmest month, and mean value of coldest month were calculated. Exactly one full year data were used in the analyses for the loggers which have been collecting brief overlapping periods. Where data were available for 3 years (three loggers), means of corresponding days were used to construct one mean annual course. The data are presented graphically in such a way that they are centered on mid-summer and start on January 1, irrespective of when the actual recording at a site commenced. Therefore, one annual course of data may be composed of data actually collected during the first half of the second year and the second half of the first year. In order to place our data collecting campaigns in the context of a longer time series of summer temperatures in that region, the May–September mean air temperatures of recent decades from the three closest weather stations are presented (Fig. S2).

## Results

**Fig. 3:**
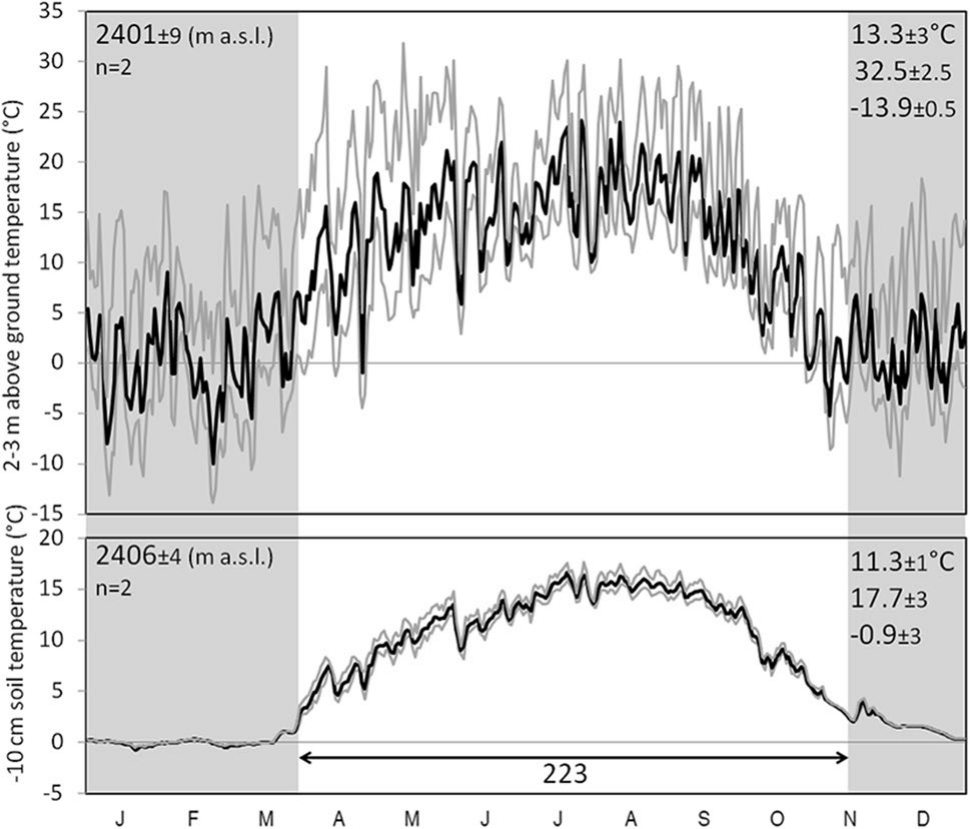
A comparison of daily air and − 10 cm soil temperature (in deep shade) for the upper forest edge in the region (*Quercus macrantherea*). Thin lines indicate hourly daily maxima and minima. Each line is the mean for two locations monitored synchronously

At regional upper limit of *Quercus macranthera* at 2400 m a.s.l., the growing season of 2014/2015 lasted 223 days, with a seasonal mean soil temperature of 11.3 °C (absolute minimum and maximum − 0.9 and 17.7 °C; Table 1; Figs. 2a, 3). It is ca. 5 K warmer than the global treeline isotherm (corresponding to a range of elevation of ca. 900 m). The diurnal and annual temperature amplitude under these trees is very low compared to air temperature (measured in the most shaded part of the canopy) and the temperatures under lower stature vegetation at all other sites (Fig. 2, S2). The tall herb-field dominated by *Prangos uloptera* in the subalpine belt (Fig. 2b), shows a three-year mean of growing season length of 163 days, with a seasonal mean soil temperature of 14.5 °C (absolute minimum and maximum temperature of − 4.8 and 30.7 °C). The coldest month is quite mild at his site (− 0.4 °C) due to snow cover.

In the high alpine zone, the snow bed community with small plants such as *Ranunculus crymophilus* (Fig. 2c) experienced a growing season of 94 days (starting on 16 July) with seasonal mean of 10 °C and the ground never below 0 °C due to snow. At the edge of these snow beds, where the open thorn-shrub plant *Astragalus iodotropis* grows (Fig. 2d), the growing season reached 96 days with a mean of 8.1 °C (absolute minimum and maximum − 0.7 and 16.8 °C). Snow cover is deep at this site as well. The flat areas at the same elevation with ca. 70 cm thorny tall herb *Cousinia multifida* (Fig. 2e) exhibit a growing season of 128 days (starting 11 June), with seasonal mean soil temperature of 11.2 °C again places with lots of snow in winter. In contrast, windswept slopes which are usually covered by thorn-cushions such as *Onobrychis cornuta* and *Acantholimon demawendicum* (Fig. 2f) have a long growing season of 153 days with a mean of 11.3 °C (− 7.6 to 27.7 °C). The longest growing season of 161 days at this elevation occurred on ridges and hill-tops inhabited by *Jurinella frigida* (Asteraceae) and some other morphologically similar species (Fig. 2g) with a mean of 10.9 °C and record temperature amplitudes from − 13.9 to 27.8 °C.

In the subnival zone, at the upper limit of the thorn-cushion community (Fig. 2h) the growing season lasted only 106 days with a mean growing season soil temperature of 6.5 °C (− 12.1 to 17.1 °C). At the limit of angiosperm life in this region at 4850 m a.s.l., where *Veronica aucheri* can reach (Fig. 2i) the growing season lasts 63 days with a seasonal mean of 4.5 °C (− 18.3 °C to 13.6 °C) and substantial diurnal soil temperature oscillations both, in the warmest and coldest months. It seems the site was snow covered only between end of May and end of June, just before the onset of the growing season, and again during September, after the growing season, given that temperature oscillations were very low during these two periods. During all other periods, these plants experience the full harshness of the climate at 4850 m elevation without protection by snow. Based on long-term records of the mean May–September air temperatures for two mountain weather stations of that region (Fig. S2), our study periods do not exhibit any significant deviations from the long term mean (± 0.3 K). The individual year data for the three years of measurements at the alpine belt (loggers b, f and g in Table 1) are shown in Fig. S3.

## Discussion

Our survey covered the thermal life conditions of plants in the Alborz from the regional upper occurrences of tree growth to the upper limit of flowering plants, over a ca. 2400 m amplitude of elevation. Over that gradient, the length of the growing season as defined by soil temperature at 10 cm depth varies from nearly 8 months at the oaks limit to 2 months at the high elevation angiosperm limit, with mean growing season soil temperatures in low stature vegetation declining from 14.5 °C to 4.5 °C. The temperatures at the regional upper limit of the Hyrcanian forest is far away from what is known to be critical for treeline globally, suggesting that this is a tree species limit, with trees missing at the potential climatic treeline. Given the precipitation data for the region (Fig. 1), this means that, much of the land above the current tree limit is treeless for reasons unrelated to climate. In the following we will discuss these temperatures in the light of the absence of treeline taxa and in terms of the ‘alpine’ nature of the treeless vegetation in comparison to the corresponding habitat types in other mountain areas.

### Tree and forest limits in the Alborz

Known as the high elevation treeline, the low temperature limit of the life-form tree is reached by more than hundred different tree species across the world (Körner 2012), whenever the mean growing season temperature falls below 6.1 °C at a growing season of at least 91-days length and a definition of days belonging to the growing season by a daily mean air temperature of at least 0.9 °C (Paulsen and Körner 2014). These limits have been validated for nearly 400 locations worldwide, provided there was a minimum of moisture (> 250 mm precipitation per year). With a mean of 6.4 °C for a 94-days length of growing season, rather similar values were obtained in an earlier, data logger based campaign like the current one, which also used a deep shade soil temperature threshold of 3.2 °C to define the boundaries of the growing season (Körner and Paulsen 2004).

In order to predict the potential treeline position in the Alborz, we employed the treeline algorithms by Paulsen and Körner (2014), but they failed, because the WorldClim data base (on which this model runs) does not account for the data of regional high elevation climate stations we had access to now, but employs lowland data like those for Tehran (231 mm of annual precipitation) which do not scale to conditions that would facilitate any tree growth at treeline. Further, the altitudinal laps rates of temperature we distilled from the closest weather stations data (Fig. S2), yielded obscure results, presumably because of very local thermal anomalies such as cold air drainage and the narrow elevation range these stations are covering. Hence, the best we could do to scale upslope from our own air temperature readings at the upper limit of the oak forest, was to apply the robust global mean laps rate obtained from many weather stations in mountains of 0.55 K 100 m^−1^ (Körner 2003), which may add some noise to our estimate.

**Fig. 4:**
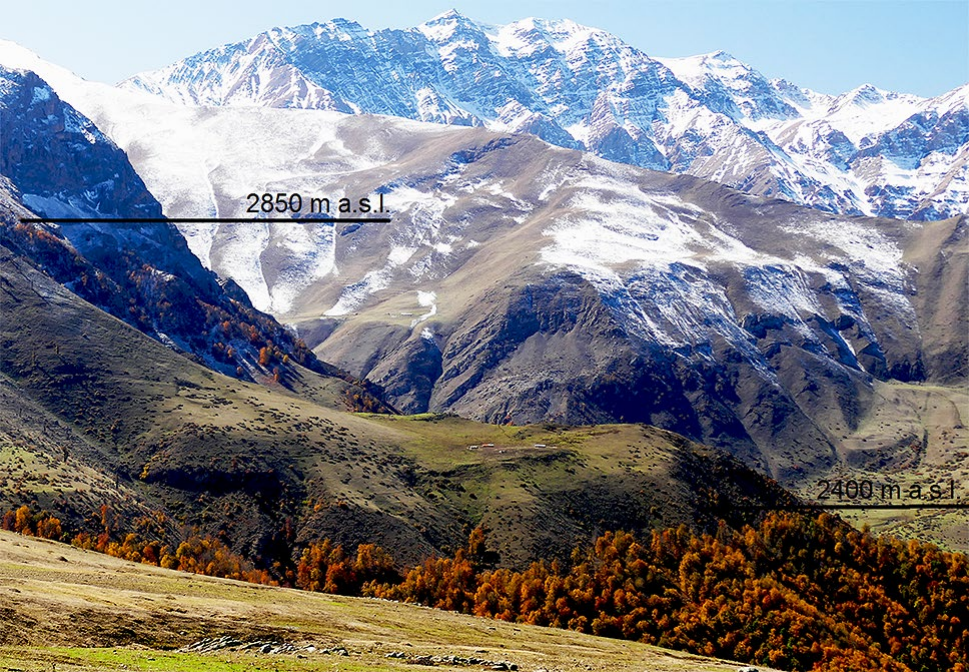
Land use (pastoralism) explains the absence of forests between 2400 m and 2850 m (the position of the uppermost tree pockets in sheltered microhabitats). Also the fragmentation of forests < 2400 m is due to land use. For the uppermost individuals of *Juniperus excelsa* see Fig. S4

A 7.5-months long growing season with a mean of 11.3 °C and at least 500 mm of annual precipitation corresponds to a cool temperate forest climate similar to what occurs over much of northern Europe lowlands, except for the prolonged summer drought of ca. 4 months. A linear regression of the treeline-latitude relationship in the northern hemisphere between (50–30°N) yields a change in treeline elevation of 130 m per degree of latitude (Körner 1998). In this case, the climatic treeline in the Alborz should be approximately 1300 m above the treeline in the Alps, (which is around 2000 m a.s.l.) that is, at around 3300 m a.s.l. If we adopt an atmospheric laps rate of temperature with altitude of 0.55 K per 100 m, we also arrive at 3300 m a.s.l., starting from the 11.3 °C at our 2400 m elevation site. Based on these estimates, land above 3300 m a.s.l. would rank as truly alpine, meaning that all sites at which we had data loggers exposed under low stature vegetation belong to the alpine belt. Open grassland below 3300 m a.s.l. is covered with Euro-Siberian *Festuco-Brometea* vegetation type (Klein and Lacoste 1994) into which alpine taxa may have invaded downslope as happened on Kilimanjaro after the destruction of the upper montane forest (Hemp 2005). *Quercus macranthera*, which forms a tree species limit of the Hyrcanian forests, potentially can reach up to 2850 m a.s.l. (Fig. 4). A bioclimatic study in this species revealed that it prefers a cool climate (Kargioglu et al. 2011) and dendrological studies showed that its growth is mainly controlled by temperature and moisture conditions during the late summer (Pourtahmasi et al. 2012; Foroozan et al. 2015). Above the *Quercus macranthera* limit, we would expect treeline taxa, such as the Euro-Siberian conifers of the Pinaceae family or representatives of the genus *Juniperus*, since that genus formed treelines in many parts of Central Asia, according to pollen records (Miehe et al. 2006). In the present flora of the Alborz, taxa of the Pinaceae family do not exist (Riedl 1965; Klein 2001). The only potential treeline species in the region is *Juniperus excelsa* which exists in some scattered areas in the southern, warmer Alborz and forms woodlands between 2000 and 3000 m a.s.l. (Ravanbakhsh et al. 2016), where precipitation gets very scarce (Fig. 1). An examination of its upper range limit using satellite images by Google Earth revealed remote, uppermost occurrences of tree-size individuals on steep slopes of Alborz at ca. 3300 m (36°30′31″ N; 54°28′07″ E; Fig. S4), which is a post-hoc confirmation of our estimates of the potential treeline position based on climate data only. The surprising convergence between (a) the latitudinal extrapolation, (b) the application of a mean altitudinal laps rate, and (c) the uppermost occurrences of *Juniperus* in the region, all arriving at ca. 3300 m, adds to the confidence in the estimated elevation of the potential treeline in the Alborz.

There are two possible explanations for the absence of trees above the current *Q. macranthera* limit. (1) Over geological periods, the Hyrcanian forests lost taxa that can grow at the humid climatic treeline. This could have occurred over periods of aridity during glacial cycles of the past million years, and these taxa never returned through natural migration corridors, otherwise used by the taxa forming the current Hyrcanian forest. Or (2), such taxa were present during the early Holocene, but were extirpated by intensive pastoral activities.

There are pollen records from the Caspian Sea and adjacent slopes covering the past 10.000 years that do not hint at any significant presence of potentially treeline forming taxa of Pinaceae family in the wider region (Leroy et al. 2013). Late Pleistocene pollen records from Lake Urmia at 1300 m a.s.l. elevation in NW Iran for the last 200,000 years reveal steppe elements and riverine taxa, with tiny traces of *Juniperus* at the peak of the last interglacial (Djamali et al. 2008). *Quercus* is quite abundant throughout the profile but mostly reflect the Zagros Anti-Taurus oak species (*Q. brantii, Q. infectoria, Q. libani*). An unpublished profile taken from Hyrcanian forests covering last 20,000 years (E. Ramezani, pers. com.) also suggests the absence of treeline taxa of Pinaceae family in this region. However, traces of *Juniperus* pollen had been recorded in all records over some past periods. This genus is known for its poor pollen deposition, hence these trace records may not represent the actual past abundance of the genus at high elevation.

Thus, it appears that the area does indeed lack suitable Pinaceae taxa that can grow at the potential treeline, and recent millennia of human land use do not seem to be the cause for that absence. An absence of treeline taxa has been found in other regions where the upper forest limits occur at far too warm temperatures, either because such hardy taxa never reached there or because of an early history of logging and burning (Pini 2002; Körner 2012). For example, upper forest edges formed by *Fagus sylvatica* in the southern Mediterranean (with a presumable land use influence; Henne et al. 2015) and *Metrosideros polymorpha* in Hawaii, and, at certain locations, the upper limits of *Nothofagus* sp. (northern part of New Zealand and Chile) and *Erica* sp. (Drakensberg escarpment, South Africa), exist at elevations beyond which cold-hardy exotic tree species could grow at higher elevation (Körner 1998).

The gap between the current Hyrcanian forest edge at ca. 2400 m elevation and those outposts that can occasionally be found up to 2850 m elevation is clearly due to pastoralism that includes severe trampling on the slopes (as illustrated in Fig. S5). That land use pressure dates back to the Neolithic period (Zeder and Hesse 2000; Diamond 2002) and has been intense in several phases during the two last millennia (Djamali et al. 2009). A cessation of pastoralism would most likely allow recruitment (now absent) and upslope expansion of *Quercus macranthera* and the associated woodland flora and help in erosion control. However, above 2850 m, there is only the above mentioned *Juniperus excelsa* that could close the treeless gap up to 3300 m a.s.l. Drought is an unlikely primary cause for that absence. This tree species is mainly distributed in the Irano-Turanian region (Gauquelin et al. 1999), which is characterized by a semi-arid, continental climate, with less clouds and thus, stronger day-time warming (Djamali et al. 2012a, b). We can only speculate why *Juniperus excelsa* is absent from treeline in the northern part of the Alborz. Dendrological studies on it in southern slopes of the Alborz show that its growth rate is sensitive to spring precipitation (Pourtahmasi et al. 2012; Foroozan et al. 2015). Yet, growth rate is not correlated with the occurrence of trees at the treeline, as evidenced by minute annual increments in the world’s highest located trees in Tibet and Bolivia at elevations > 4800 m in semi-arid climates (Körner 2012). Supposedly, the very slow growth rate *per se* does not exclude mature *Juniperus excelsa* from treeline, yet, once such stands become destroyed this species exhibits a very low capacity to regenerate (Akhani 1998). In more humid areas, pathogens may play a restrictive role. So, our best guess is that it is the combination of past, destructive land use and poor regeneration potential (related to intrinsic slow growth) that explains the absence of *Junipers* from the potential treeline in the northern Alborz. This hypothesis awaits to be tested by plantations (protected from browsing and trampling) in the northern Alborz between 3000 and 3300 m a.s.l.

### Alpine vegetation in the Alborz

The data for low stature vegetation above the regional tree species limit illustrates that a dense ground cover such as the sheltered herbfields at 3475 m elevation can trap solar heat so that soils actually became warmer across the season (mean 14.5 °C) than they are at 1000 m lower elevation under trees. The dominant species of this vegetation type, *Prangos uloptera*, an ephemeral herb, loses the above ground parts in the middle of the hot growing season due to overgrazing and animal trampling. The location of our logger in that type of vegetation was on a south facing slope, which may have contributed to the high temperatures.

Snow melt gradients in higher alpine regions permit investigating the relationship between climate and plant communities over different time scales (Galen and Stanton 1995). On steep slopes, ridges and depressions, the interplay between exposure and vegetation is leading to mosaics of life conditions (Körner et al. 2003) clearly associated with snow duration as evidenced by our soil temperature data. As expected, the growing season is shortest in snow beds (94 days) and longest on ridges (161 days) at similar elevation. In the high alpine zone, snow beds are inhabited by small rosette species (hemicryptophytes). With increasing length of the growing season, the size of plants increases, but on the ridges small rosette plants are still dominant. This supports previous results that showed that sites with an early melt regime host tall herb fields, and sites with a late melt-out typically attract plant communities with taxa of shorter seasonal life cycles (Jonas et al. 2008). The dry mid-summer does not allow alpine plants to be active after August. Therefore, a temperature-only defined growing season, does not mean that plants can use that entire period.

The soil temperature during the growing season in snow bed communities can be higher than under open thorn-shrub communities because the canopy of *Astragalus iodotropis* keeps the ground shaded. In steep slopes covered by thorn-cushion communities, the ground is not well protected by snow during the non-growing season and soils are freezing. But the shrub is still providing shelter to other taxa (facilitation; Cavieres et al. 2014). Because of strong winds, exposed ridges and hill-tops are always free of snow, even during the coldest months, thus, causing winter temperatures to drop to very low values. The same locations get warmer than other places during the growing season due to strong insolation. *Jurinella frigida* seems to be well adapted to both, the heat/drought in summer and the severe freezing in winter.

At the upper limit of vascular plant life at 4850 m a.s.l., the growing season is very short and cold. *Veronica aucheri*, a small hemicryptophyte endemic to the Central Alborz is the only species found at such high elevation. In the European Alps, the highest climbing species is *Saxifraga oppositifolia*, a small cushion plant, also widely distributed in the Arctic, that is reaching 4505 m elevation and was found to operate at a 45 day season and a mean of 2.6 °C (Körner 2011).

Overall, the seasonal ground temperatures reveal that topography plays a very important role for thermal life conditions. Although season length may vary from year to year, 3–6 months appear to be the range over which the alpine communities operate in the Alborz. Snow cover exerts a strong moderating role on ground freezing. The smallest annual amplitude of − 10 cm soil temperature was 17.5 K in snowbeds at 3693 m a.s.l., and the largest amplitude was 41.7 K on a nearby ridge at 3710 m a.s.l. (Fig. 2).

Compared to other alpine ecosystems examined with the same protocol (Körner et al. 2003), the Alborz alpine vegetation at 36° N experiences a growing season mean of 10.3 °C for the 5 sites between 3690 and 3710 m a.s.l. (ca. 400 m above the estimated potential treeline position), which is ca. 1 K warmer than the 9.2 °C mean for the 5 European alpine sites examined at ca. 200 m above the local climatic treeline between 44° and 47° N. The warmest month (August) mean of 15.4 °C for the Alborz is, however clearly warmer than the 11.7 °C for the Alps, a likely result of the clear summer skies in the Alborz, compared to the often rather cloudy weather in European mountains. Surprisingly, the length of the growing season defined by the same temperature thresholds is shorter rather than longer for the five Alborz sites than for the five sites in the Alps (126 compared to 153), but if we exclude the two snow bed sites at 3690 and 3693 m a.s.l., the resulting mean 147 days of season duration for more exposed sites is similar, particularly when unresolved year to year variation is not accounted for. The much higher temperatures at the 3475 m a.s.l. site come close to what had been observed in the Sierra Nevada of Spain and on Mount Etna at 37° N, regions, that also lack trees at the local potential climatic treeline (Körner et al. 2003). The annual absolute minima (− 0.1 to − 13.9 °C) and maxima (16.8 to 27.8 °C) of soil temperature across the sites in the Alborz are similar for the minima, but cover wider ranges for maxima than have been observed in the Alps (− 1.4 to − 15.2 °C and 13.5 to 19.2 °C) owing to the more continental climate and steeper solar angle. Given all these comparisons refer to − 10 cm soil temperature, the extremes in the Alborz would be even more pronounced at ground surface and small stature plant level.

## Conclusion

The data-sets for the regional forest limit and from different alpine and subnival elevations and topographic positions, provide an overview of the thermal growth conditions between the edge of the Hyrcanian forests and the edge of angiosperm life between 2400 and 4850 m a.s.l. in the Central Alborz. From the data collected here, we conclude: (1) Seasonal mean temperatures at the upper limit of the Hyrcanian forests are 5 K warmer than expected for a natural climatic tree limit (the treeline). The absence of trees up to 2850 m (450 m below the estimated treeline at 3300 m a.s.l.) is best explained by millennia of detrimental land use practices. The absence of trees between 2850 m and 3300 m (above the *Quercus macrathera* limit) is due to the regional lack of suitable taxa (e.g. the absence of Pinaceae species) which is also supported by pollen records. However, the existence of isolated individuals of *Juniperus excelsa* up to 3300 m a.s.l. on steep dry slopes suggests that this species could potentially fill that gap, but due to its poor regeneration it could not recover from past destruction either by wild fires or land use (or both). Given the ca. 550 mm of annual precipitation, drought is an unlikely cause for the absence of drought adapted trees. Also rain-fall seasonality (long dry summer) does not explain the absence of an aridity-adapted species such as *Juniperus excelsa* from high elevation terrain that is more humid than where its relicts are currently found. At similar precipitation, the low elevation, thermophilous eastern Mediterranean forests cope with 6–8 rainless month and summer temperatures up to 40 °C (Sarris et al. 2011). The land above 3300 m a.s.l. is to be rated as truly alpine, the treeless terrain between 2850 and 3300 m a.s.l. belongs to the subalpine belt, which became inhabitable for some alpine taxa because of the absence of a forest cover. (2) The alpine vegetation of the Alborz experiences similar growing season temperatures and growing season duration as had been found in the Alps at 10° higher latitude. Although this region is combined with a dry, nearly cloudless summer and thus, warmer temperature maxima, due to strong insolation and reduced evaporative cooling in the Alborz. The dry summer requires deep plant rooting, with drought adding a stress component (to be explored and quantified) that is absent in the Alps. (3) Topography and snow melt regimes exert an important role in shaping the alpine vegetation. At the same elevation and same place, across a few meters distance, the growing season can be less than 3 months (with hardly any winter freezing) or more than 5 months (with exposure to severe freezing), which plays an important role for plant species composition and the dominance of certain physio- and morphotypes of plants.

## Electronic supplementary material

Below is the link to the electronic supplementary material.

**Table S1** Vegetation relevés for the uppermost patches of the Hyrcanian forest in sheltered locations at the north-slope of the Central Alborz (Klein and Lacoste 1989) (XLSX 43 KB)

**Fig. S2** A long time series of summer temperatures (May-September mean air temperatures) from three weather stations close to study area. See the location and climate diagrams of the stations in Fig. 1 (Tehran: 35° 42’ N, 51° 19’ E; Abali: 35° 45’ N, 51 °53’ E; Siyahbisheh: 36° 15’ N, 51° 18’ E). For comparison we added the longer-term data series for Tehran, a low elevation station. All three sites show a warming trend, best captured by the Tehran station (including an urbanization effect since the early records). We have no explanation why one of the two mountain station (Abali) shows a minimum in 2012, whereas the other one and Tehran do not. Presumably some local cold air drainage phenomena played a role (DOCX 48 KB)

**Fig. S3** Individual year records for three years of − 10 cm soil temperature for loggers b, f, g (Table 1). Seasonal mean temperature, absolute minimum and maximum are shown in the top right corner (DOCX 306 KB)

**Fig. S4** The uppermost occurrences of tree-size individuals of *Juniperus excelsa* on steep slopes at ca. 3300 m a.s.l. explored by using satellite images of Google Earth (DOCX 2150 KB)

**Fig. S5** Dramatic effect of cattle grazing and trampling in the upper montane belt of the Hyrcanian region. The land shown here at ca. 2500 m a.s.l. could carry a closed forest based on our climate data (DOCX 941 KB)
