# Supplementary figures and images for: A bioclimatic characterization of high elevation habitats in the Alborz mountains of Iran
Source: Alp Bot. 2018 Feb 6;128(1):1–11. doi: 10.1007/s00035-018-0202-9 (PMC5856880; doi:10.1007/s00035-018-0202-9)

Fig. S2


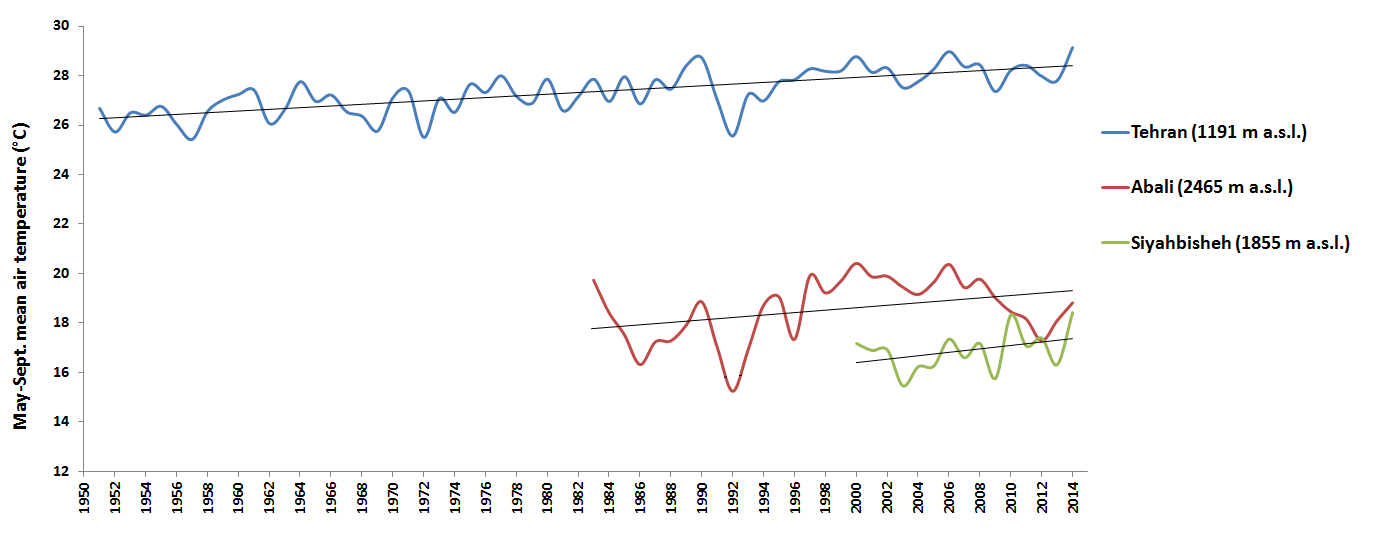

Supplement: Supplementary file 2 — Fig. S2 A long time series of summer temperatures (May-September mean air temperatures) from three weather stations close to study area. See the location and climate diagrams of the stations in Fig. 1 (Tehran: 35° 42’ N, 51° 19’ E; Abali: 35° 45’ N, 51 °53’ E; Siyahbisheh: 36° 15’ N, 51° 18’ E). For comparison we added the longer-term data series for Tehran, a low elevation station. All three sites show a warming trend, best captured by the Tehran station (including an urbanization effect since the early records). We have no explanation why one of the two mountain station (Abali) shows a minimum in 2012, whereas the other one and Tehran do not. Presumably some local cold air drainage phenomena played a role (DOCX 48 KB) [file 35_2018_202_MOESM2_ESM.docx]

Fig. S3


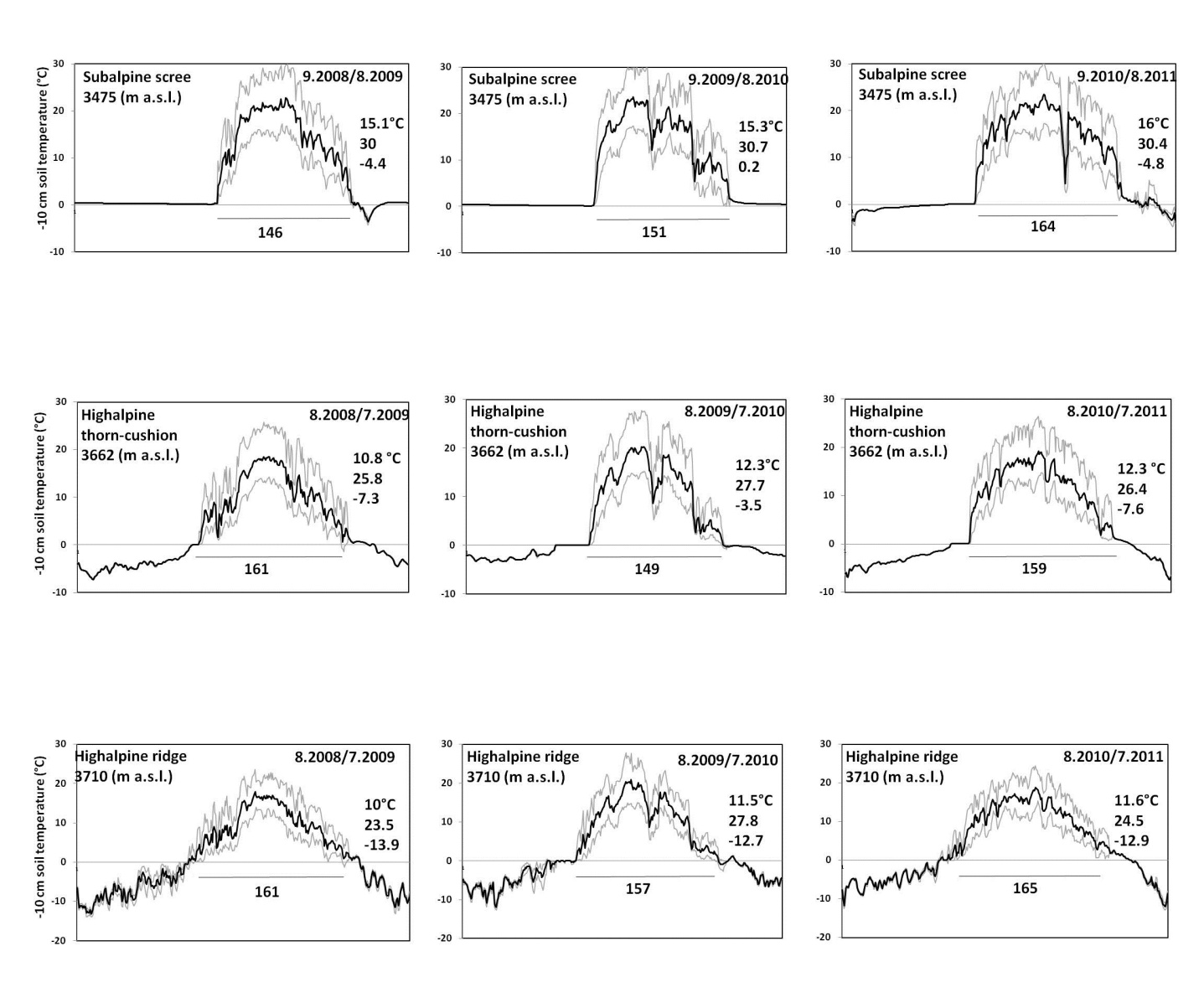

Supplement: Supplementary file 3 — Fig. S3 Individual year records for three years of − 10 cm soil temperature for loggers b, f, g (Table 1). Seasonal mean temperature, absolute minimum and maximum are shown in the top right corner (DOCX 306 KB) [file 35_2018_202_MOESM3_ESM.docx]

Fig. S4


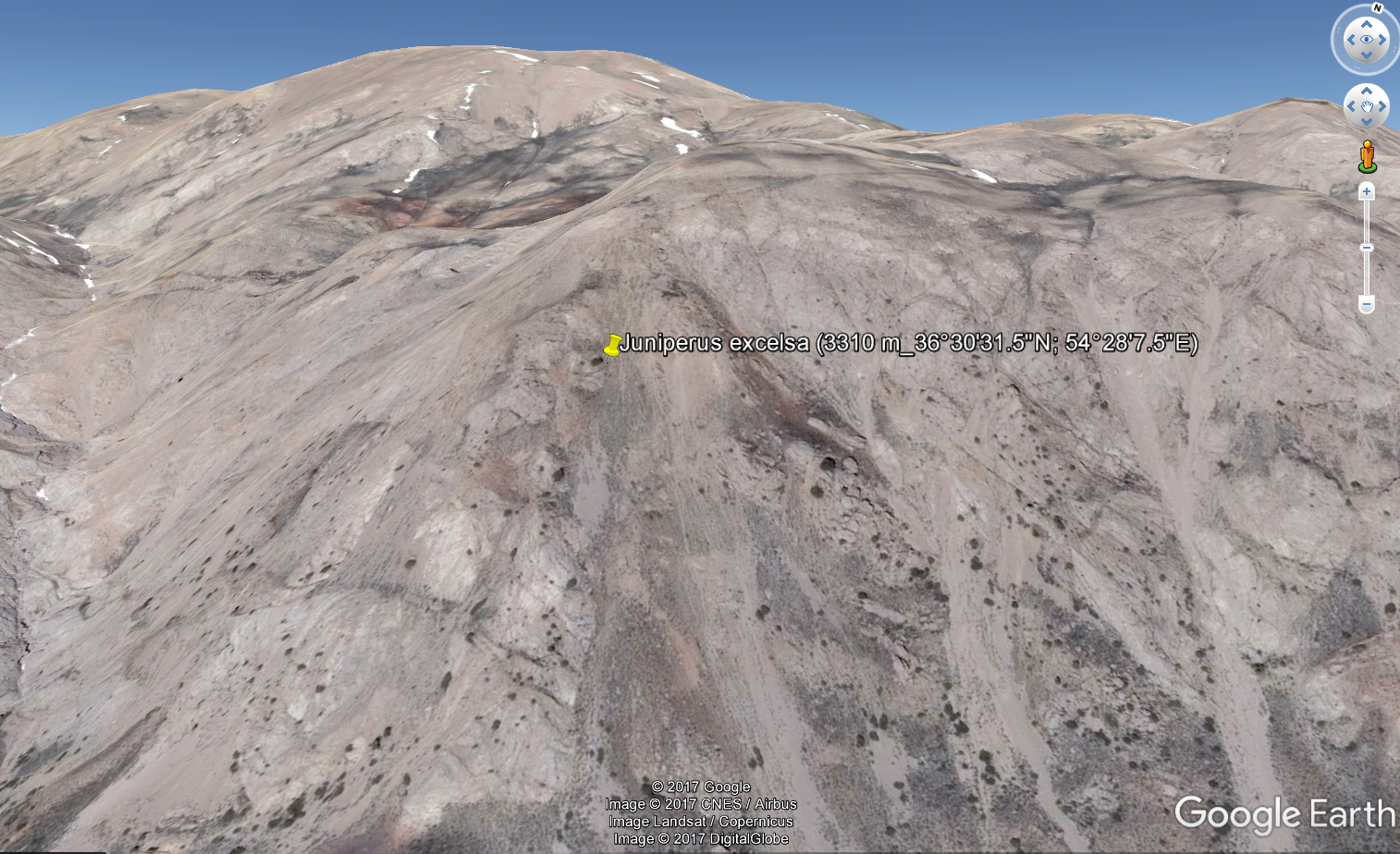

Supplement: Supplementary file 4 — Fig. S4 The uppermost occurrences of tree-size individuals of Juniperus excelsa on steep slopes at ca. 3300 m a.s.l. explored by using satellite images of Google Earth (DOCX 2150 KB) [file 35_2018_202_MOESM4_ESM.docx]

Fig. S5


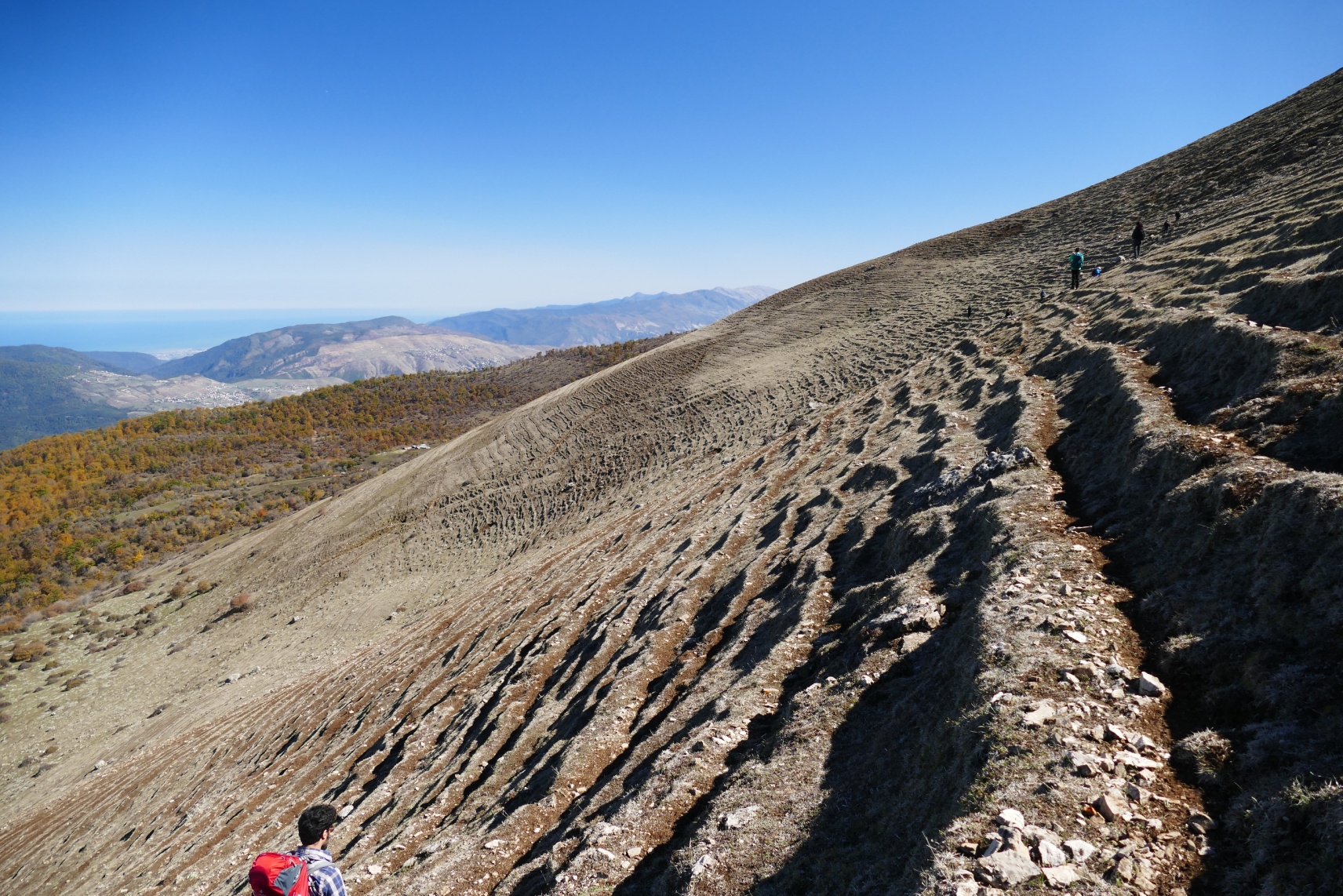

Supplement: Supplementary file 5 — Fig. S5 Dramatic effect of cattle grazing and trampling in the upper montane belt of the Hyrcanian region. The land shown here at ca. 2500 m a.s.l. could carry a closed forest based on our climate data (DOCX 941 KB) [file 35_2018_202_MOESM5_ESM.docx]
